# How Do You Feel Now? The Salience Network Functional Connectivity in End-Stage Renal Disease

**DOI:** 10.3389/fnins.2020.533910

**Published:** 2020-11-11

**Authors:** Runyue Hu, Lei Gao, Peina Chen, Baolin Wu, Xiaoyan Wu, Haibo Xu

**Affiliations:** ^1^Department of Radiology, Zhongnan Hospital of Wuhan University, Wuhan, China; ^2^Department of Nephrology, Zhongnan Hospital of Wuhan University, Wuhan, China; ^3^Department of Nephrology, Shantou Central Hospital, Affiliated Shantou Hospital of Sun Yat-sen University, Shantou, China

**Keywords:** cognitive impairment, linear support vector machine, classifier, mediation analysis, anemia

## Abstract

**Objective:**

The network connectivity basis of cognitive declines in end-stage renal disease (ESRD) remains unclear. A triple-network model of the salience (SN), executive control, and default mode networks has been suggested to be critical for efficient cognition. Here, we aimed to test the hypothesis that SN may play a role in cognitive impairment in patients with ESRD.

**Materials and Methods:**

We investigated functional connectivity (FC) alterations within the SN between 43 ESRD patients (19 females/24 males, 46 ± 10 years) and 43 healthy controls (HC) (19 females/24 males, 47 ± 10 years), and performed linear support vector machine (LSVM) analysis on significant FC pairs within the SN to discriminate the two groups, and tested the accuracy of the classifier. Association and mediation analyses were conducted among the significant FC pairs within the SN nodes, clinical indicators, and neuropsychological tests scores.

**Results:**

We identified significant between-group FC pairs within the SN and fairly good classification efficiency with significant accuracy (72.09%, *p* < 0.001). We found that FC between the right supramarginal gyrus and right anterior insula (AISL) was positively correlated with MoCA (*r* = 0.4010, *p* = 0.008); FC between the dorsal anterior cingulate cortex (dACC) and left AISL was positively correlated with the level of hemoglobin (*r* = 0.4979, *p* < 0.001). Mediation analysis found that the indirect effect of hemoglobin on forward digit span test scores via the FC between the dACC and right AISL (*p* < 0.05).

**Conclusion:**

Disrupted SN connectivity may help explain cognitive declines in ESRD patients and act as a potential early biomarker. Moreover, the SN connectivity may interact with anemia to promote cognitive impairment.

## Introduction

End-stage renal disease (ESRD) is a final condition of kidney function decline with a glomerular filtration rate (GFR) of less than 15 mL/min/1.73 m^2^ (Webster et al., 2017). With the advancement of medical treatment, ESRD has changed from a once fatal disease to a now chronically controllable state. But ESRD patients still suffer a higher risk of developing cognitive impairment, and its pathophysiology remains unclear (Bugnicourt et al., 2013).

A proposed kidney-brain axis is the pathophysiologic interaction between renal damage and brain function, putatively thought to be a significant cause of cognitive deteriorations in ESRD patients (Bugnicourt et al., 2013). ESRD is associated with significant brain injury, for example high levels of uremic toxins (UT) in the blood and brain (De Deyn et al., 2009; Watanabe et al., 2014), prevalent anemia (Kurella Tamura et al., 2011), high incidence of vascular risk factors, and chronic inflammation, which may together promote cerebral small vascular injury, endothelial dysfunction, neurodegenerative alterations, and disturbances of the autonomic nervous system (Kooman et al., 2014; Toyoda, 2015). However, it is unclear how these risk factors induced by renal dysfunction influence brain function, especially cognition.

Brain structural and functional imaging provides new insights into ESRD-related cognitive dysfunction. Structural studies reported that ESRD patients had reduced gray matter volume and white matter integrity, including the bilateral insula and anterior cingulate cortex (ACC), which was correlated with decreased kidney function and cognitive decline (Zhang et al., 2013; Sedaghat et al., 2015; Mu et al., 2018a). Resting-state functional connectivity (FC), in contrast, may sensitively reflect the energy demands associated with synaptic signal transmission (Tomasi et al., 2013). For example, ESRD patients with neuropsychological impairments demonstrated widespread decreased network connectivity, including the default mode network (DMN), executive control network (CEN) and salience network (SN), which can be recovered early after kidney transplantation resulting in cognitive improvement (Chen et al., 2018). The triple-network model of SN, CEN, and DMN have turned out to be particularly important for maintaining higher cognitive function, and the alteration of FC in the triple network model was observed in subjects with Alzheimer’s disease, mild cognitive impairment, and high risk subjects (Joo et al., 2016). The SN, including the medial frontal cortex, dorsolateral prefrontal cortex (dlPFC), dorsal anterior cingulate cortex (dACC), anterior insula, thalamus and cerebellum lobule VI, and the adjacent crus I, is engaged in identifying and responding to salient stimuli, recruiting relevant large-scale functional networks, and especially coordinating CEN and DMN for cognition control (Menon, 2011; Uddin, 2015; Levar et al., 2019). The interrupted FC in the SN may lay the foundation for cognitive decline and become neurological markers (Chand et al., 2017). Though recent ESRD-related research have found structural changes and abnormal FC in the SN from a whole brain perspective, little is known about the relationship between the altered SN connectivity and cognitive impairment and kidney-brain axis in patients with ESRD, and whether the altered SN connectivity can be used to distinguish ESRD patients from healthy controls (HC).

We hypothesized that SN connectivity is altered in ESRD and can be discriminated from HC, and we further investigated the relationship between the altered FC within the SN, cognitive function, and ESRD. First, we used resting-state functional MRI (rs-fMRI) to measure the alteration of FC within the SN nodes. Second, association analysis was conducted between significant SN connectivity and clinical indicators, and cognitive tests in the ESRD group. Third, mediation analysis was used to investigate the underlying relationship between cognitive impairment, SN dysconnectivity, and ESRD by examining the direct and indirect effects between multiple risk factors of the kidney-brain axis and cognitive function. In order to confirm if the significant SN connectivity can distinguish between the ESRD group and HC group, we used linear support vector machine (LSVM) analysis based on statistically significant FC pairs in the SN, tested the accuracy of the classifier, and calculated the contributing weights of each significant FC pair.

## Materials and Methods

### Participants

This study was approved by the Ethics Committee of the Zhongnan Hospital of Wuhan University, and written informed consent was obtained from all participants prior to the experiment. The inclusion criteria for ESRD were as follows: (1) clinical diagnosis of ESRD, with estimated glomerular filtration rate ≤15 mL/min/1.73 m^2^, and treatment of maintenance hemodialysis; (2) age 18–65 years, right-handed; and (3) without MRI contraindications (metallic implants in body, fever, claustrophobia, and pregnant woman). The exclusion criteria were: (1) obvious brain lesions such as intracranial masses and cerebral infarction; (2) neurologic, psychiatric disorders, malignant tumor; (3) history of drug or alcohol abuse; (4) other systemic dysfunctions such as the heart and liver, or organ transplantation; (5) acute cardiovascular or cerebrovascular diseases, acute infections; (6) motor or sensory deficits; and (7) severe head movement during scanning (head movement exceeding 3 mm or 3°) or unable to cooperate with the scanner.

The inclusion criteria for HC included: (1) age 18–65 years, right-handed; (2) the mini-mental state examination (MMSE) score >24; and (3) without MRI contraindications. The exclusion criteria included: any psychiatric, neurologic, immune, metabolic disease, comorbid medical conditions that affect cognitive function, a family history of dementia or neuropsychiatric disorders, diabetes, liver or kidney diseases, cardiovascular or cerebrovascular diseases, acute infections, any history of abuse of drugs or alcohol, smoking, or major sensory deficits, and head movement exceeding 3 mm or 3°during scanning.

### MRI Data Acquisition and Analysis

MRI were obtained on a 3.0 T magnetic resonance scanner (Prisma, Siemens, Erlangen, Germany) equipped with a standard Siemens 64-channel head coil. Conventional T1-weighted, T2-weighted, and T2-weighted-Fluid-Attenuated Inversion Recovery (FLAIR) images were scanned to rule out artifacts and obvious brain lesions. Blood oxygenation-dependent (BOLD) rs-fMRI data were acquired with Gradient-Recalled Echo-Planar Imaging (GRE-EPI) (repetition time/echo time = 2000 ms/30 ms, matrix = 64 × 64, slice thickness = 4 mm, flip angle = 78°, a total of 240 volumes and 8 min in length). Anatomic images were acquired in high-resolution anatomic T1 Magnetization Prepared RApid Gradient Echo (T1-MPRAGE) sequence: repetition time/echo time = 2000 ms/2.30 ms, matrix = 256 × 256, flip angle = 8°, sagittal 176 slices, slice thickness = 1 mm.

The rs-fMRI data were preprocessed using Data Processing and Analysis for (Resting-State) Brain Imaging (DPABI) (Yan et al., 2016)^1^, Statistical Parametric Mapping (SPM8^2^), and MATLAB^3^. Briefly, the main steps included: (a) the removal of the first 10 functional volumes, (b) slice timing, (c) realignment and head motion correction, subject would be excluded if their functional MRI scan head movement was more than 3 mm or 3°, (d) co-registration between the T1 anatomical images and the functional volumes, (e) the co-registered T1 images were then segmented into gray matter, white matter, and cerebrospinal fluid (CSF) using new segment and Diffeomorphic Anatomical Registration Through Exponentiated Lie Algebra (DARTEL) (Ashburner, 2007), (f) normalization of the functional volumes into the standard Montréal neurological institute (MNI) 152 space (resliced into 2 × 2 × 2 mm^3^), (g) linear detrending, (h) regression of nuisance (mean signals from the white matter, the CSF, and the Friston 24 head motion parameters), by default, we did not remove global signals, and (i) temporal bandpass filtering (<0.1 Hz).

We obtained frame-wise displacement (FD) parameters from the head motion parameters, then performed two-sample independent *t*-tests on the mean FD power to test if there were statistical differences between the ESRD and HC group, meanwhile we reported the mean value of each group and the statistical values. If there was statistical difference, we would continue to remove FD parameters as covariates.

**FIGURE 1:**
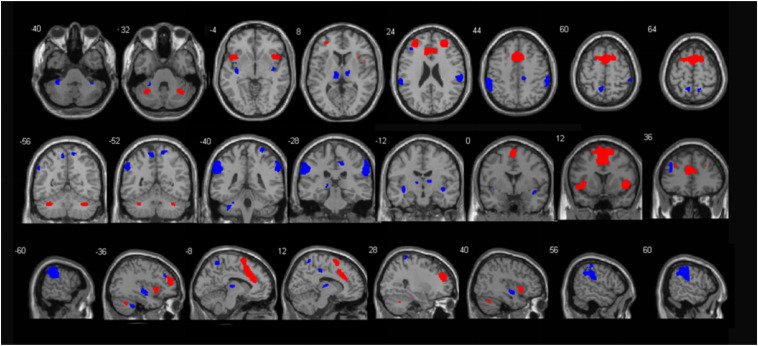
Illustration of the anterior (red) and posterior (blue) SN regions. These templates were created by Shirer and colleagues (http://findlab.stanford.edu/functional_ROIs.html).

Seed regions of the SN. We used well-validated regions of interest (ROI) that encompassed 7 anterior and 12 posterior SN nodes available at^4^, which were isolated by independent component analysis (Shirer et al., 2012), as illustrated in Figure 1 and Supplementary Table S1.

The FC analysis was performed using the DPABI. ROI to ROI correlations based on the low-frequency (<0.1 Hz) filtered BOLD signal were computed and resulting in a 19 × 19 correlation matrix. This matrix was then z-shifted individually.

### Network-Based Statistic (NBS)

The NBS is a graph analog of cluster-based statistical methods for mass univariate testing on all pixels in an image. We used NBS Connectome version 1.2^5^ to obtain significant FC between ESRD patients and the HC group. The main steps included: first, two sample *t* tests were performed on FC between the two groups; second, the FC pairs with a test statistic value exceeding 3.1 were admitted to a set of supra-threshold connections; third, topological clusters among the supra-threshold FC were identified. Finally, one-sided family wise error rate (FWER)-corrected *p*-values were calculated for each FC using permutation tests (*N* = 5000) between the ESRD group and HC group (FWE corrected, *p* < 0.05). For each permutation, the first three steps of the NBS were repeated on the permuted data. According to the test results between the two groups, the ROI to ROI matrix was transferred into a matrix where the significant FC pairs were set to 1 and others were set to 0. The matrix was multiplied by the ROI matrix of each subject to obtain the corresponding functional connection *z* value. The *z* value of each significant FC pair was extracted from the FC matrix of all the subjects.

### Linear Support Vector Machine (LSVM) Based Classification

The LSVM-based classification was conducted using the LIBSVM toolbox^6^. We constructed a class vector comprising either “+ 1” (ESRD) or “-1” (HC). Each of the significant between-groups FC pairs via the NBS was converted into a feature vector. The LSVM used significant FC (*z* values) that had been previously sorted into groups as input to build the classifier that best separated the groups matching them with the known class labels by a leave-one-out cross validation (LOOCV) strategy. Then the LOOCV strategy was also used to evaluate the classification performance with 5,000 permutations (Dai et al., 2012). Thus accuracy (the proportion of subjects classified correctly), sensitivity (the proportion of ESRD classified correctly), specificity (the proportion of HC classified correctly), positive predictive value (PPV: the proportion of correct ESRD predictions), and negative predictive value (NPV: the proportion of correct HC predictions) were computed to quantify our classification performance. Area under curve (AUC) was calculated to evaluate effect size. Finally, the discriminative weight of each significant FC pair (the greater absolute value of weight indicated the greater contribution to the classifier) was defined as the average of their absolute weight across all folds (feature selection would be different in different LOOCV folds for different data sets) to evaluate the contribution of each significant FC pair in the process of building the classifier (Cui et al., 2016).

### Neuropsychological and Laboratory Examinations

The ESRD patients completed the laboratory and neuropsychological tests 1 day prior to the MRI scan. The HC only underwent neuropsychological tests around their MRI scans. The laboratory examinations included tests for serum urea nitrogen, serum creatinine, triglyceride, cholesterol, low-density lipoprotein, and hemoglobin. Standardized neuropsychology tests included the Montreal cognitive assessment (MoCA) scale, trail making test A (TMT-A), trail making test B (TMT-B), forward digit span task (FDST), backward digit span task (BDST), symbol digit modalities test (SDMT), Hamilton anxiety scale (HAMA), and the Hamilton depression scale (HAMD).

### Association Analysis

By using SPSS 22.0, we first tested the normality of distribution of the neuropsychological test scores and clinical variables in the ESRD group. Then Pearson’s correlations were conducted between the behavioral variables (MoCA, TMT-A, FDST, BDST, SDMT, HAMA, and HAMD) and the significant FC pairs within the SN (z scores); and Spearman’s rank-order correlations were conducted between TMT-B and the significant FC pairs within the SN in the ESRD patients. Spearman’s rank-order correlations were conducted between the significant FC pairs and clinical variables (triglyceride, low-density lipoprotein); and Pearson’s correlations were conducted between the significant FC pairs and clinical variables (serum urea nitrogen, serum creatinine, cholesterol, and hemoglobin) in the ESRD patients.

### Mediation Analysis

To investigate the underlying mechanism of cognitive decline based on multiple risk factors in the kidney-brain axis (clinical variables included serum urea nitrogen, serum creatinine, triglyceride, cholesterol, low-density lipoprotein, and hemoglobin), we performed mediation analysis to examine the effects of these clinical variables on the cognitive behavior and significant between-groups FC of the SN (significant between-groups FC pairs by NBS) in ESRD patients and test whether the significant FC could mediate the effects of these clinical variables on neuropsychological tests (MoCA, TMT-A, TMT-B, FDST, BDST, SDMT, HAMA, and HAMD) by using model 4 of the PROCESS software (Hayes, 2012) (v.3.4) implanted in SPSS, with age, gender, and education as covariates. The test included five steps: (1) *c*: the total effect of the clinical variables (*X*) on cognitive impairment (*Y*) by regressing *Y* on *X* alone (*Y* = *i* + *cX* + *e*); (2) *a*: the effect of *X* on significant FC within the SN (*M*) by estimate *M* from *X* (*M* = *i_M_* + *aX* + *e*_M_); (3) *b*: the effect of *M* on *Y* after controlling *X* (*Y* = *i_Y_* + *c’X* + *bM* + *e*_Y_); (4) *c’*: the direct effect of *X* on *Y* after controlling *M* (*Y* = *i_Y_* + *c’X* + *bM* + *e*_Y_); and (5) *a* × *b*: the indirect effect of *X* on *Y* by testing whether the relationship was significant after controlling *M*, so *c* = *c’* + *a* × *b*. The above mentioned i.e., *i*_M_, *e_M,_ i_Y_*, and *e*_Y_ stood for parameters from the corresponding equation (Hayes, 2012). The total effects, direct effects, and indirect effects were considered significant when the 95% confidence intervals (CI) did not contain zero in the 5,000 bootstrap samples corrected. Mediation effects may exist when indirect effects were significant.

### Statistics

The clinical and behavioral variables were compared between the two groups using a two-sample independent *t*-test for continuous variables and a Chi-square test for gender in SPSS 22.0 (SPSS Inc., Chicago, IL), with a obviously significant level set at *p* ≤ 0.001.

## Results

### Demographic and Behavioral Results

Compared with the HC group, the ESRD group demonstrated significantly poorer global cognition measured by MoCA (*p* < 0.001), poorer working memory and attention measured by FDST (*p* < 0.001) and BDST (*p* = 0.001), and poorer processing speed measured by SDMT (*p* = 0.001) and TMT-A (*p* = 0.001). Additionally, the ESRD patients exhibited significantly high depression scores, as measured by HAMD (*p* < 0.001). There was no difference in gender, age, education, or mean FD power between the two groups (*p* > 0.05). Demographics and behavioral results are summarized in Table 1.

**TABLE 1:** Demographic and behavioral results.

	HC (*n* = 43) mean (SD)	ESRD (*n* = 43) mean (SD)	*t* value	Cohen’s d	95% Confidence interval	*p* value
Age, y	46 (10)	47 (10)	−0.084	0.018	−0.441–0.405	0.933^a^
Education, y	10.8 (4.1)	9.6 (3.0)	1.515	0.327	−0.100–0.751	0.134^a^
Gender	24M/19F	24M/19F	–	–	–	>0.99^*b*^
FD	0.1754 (0.1246)	0.1884 (0.1332)	−0.466	0.101	−0.523–0.323	0.642^a^
MoCA	26 (3)	23 (5)	3.927	0.847	0.403–1.286	<0.001^a^
HAMD	4.6 (3.9)	11.5 (7.4)	−5.376	1.159	−1.614–−0.699	<0.001^a^
HAMA	5.1 (3.5)	8.3 (7.1)	−2.702	0.583	−1.013–−0.149	0.008^a^
SDMT	50 (14)	41 (10)	3.386	0.730	0.291–1.165	0.001^a^
FDST	6.9 (1.5)	5.3 (1.4)	4.750	1.024	0.572–1.472	<0.001^a^
BDST	5.9 (1.6)	4.9 (1.2)	3.302	0.712	0.274–1.146	0.001^a^
TMT-A (seconds)	56 (27)	76 (29)	−3.438	0.742	−1.177–−0.302	0.001^a^
TMT-B (seconds)	68 (35)	97 (55)	−2.913	0.628	−1.060–−0.193	0.005^a^
BUN (in mol/L)	N/A	19.1 (10.2)	–	–	–	–
Cr (in μmol/L)	N/A	677.4 (294.1)	–	–	–	–
Triglyceride (in mmol/L)	N/A	1.9 (1.2)	–	–	–	–
Cholesterol (in mmol/L)	N/A	4.2 (1.0)	–	–	–	–
LDL (in mmol/L)	N/A	2.3 (0.7)	–	–	–	–
RBC (× 1012/L)	N/A	3.0 (0.8)	–	–	–	–
Hemoglobin (g/L)	N/A	89.7 (22.3)	–	–	–	–
Hematocrit (%)	N/A	27.5 (7.2)	–	–	–	–

*^*a*^:two-sample *t*-test. ^*b*^:Chi-square test. FD, mean power of frame-wise displacement; MoCA, Montreal cognitive assessment scale; HAMD, Hamilton depression scale; HAMA, Hamilton anxiety scale; SDMT, symbol digit modalities test; FDST, forward digit span task; BDST, backward digit span task; TMT-A, Trail Making Test A; TMT-B, Trail Making Test B. BUN, Serum urea nitrogen; Cr, Serum creatinine; LDL, Low-density lipoprotein; RBC, red blood cell; M, male; F, female.*

### Network-Based Statistical Results

**FIGURE 2:**
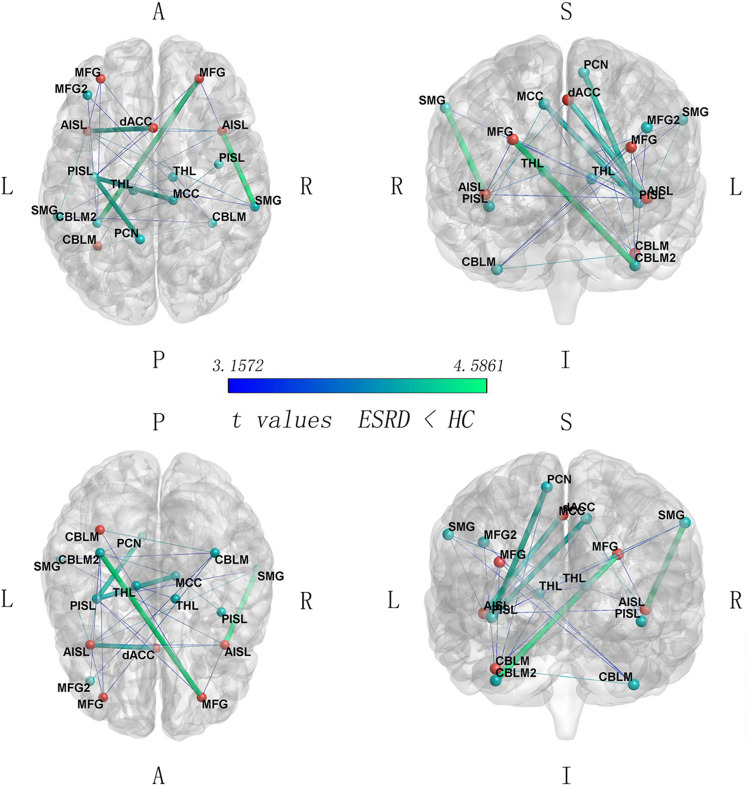
Between-group comparison on SN connectivity (ESRD < HC, FWE corrected *p* < 0.05). Red nodes, the anterior SN regions; green nodes, the posterior SN regions. SMG, supramarginal gyrus; CBLM, cerebellum_6; CBLM2, cerebellum_6(2); MFG, middle frontal gyrus; MFG2, middle frontal gyrus (2); AISL, anterior insula; PISL, posterior insula; PCN, precuneus; THL, thalamus; MCC, middle cingulate cortex; L, left; R, right; A, anterior; P, posterior; S, superior; I, inferior. The five thicker edges represent the FC links with the 20% of *t* values.

Compared with HC group, some FC pairs of the SN were significantly decreased in the ESRD patients (Figure 2 and Table 2), especially the FC between the dACC and the left anterior insula (AISL), between the right AISL and the right supramarginal gyrus (SMG), between the left posterior insula (PISL) and the right middle cingulate cortex (MCC) and the left precuneus (PCN), and between the left cerebellum lobule VI (CBLM-VI) and the right middle frontal gyrus (MFG), that the *T* values ranked the top 20% in the significant FC pairs within the SN.

**TABLE 2:** Between-group comparison on SN connectivity (ESRD < HC, FWE corrected *p* < 0.05).

Functional connectivity	*T* values	Functional connectivity	*T* values
Anterior insula_R to supramarginal_R	4.59	Anterior insula_R to supramarginal_L	3.63
Frontal_mid_R to cerebellum_6_L2	4.55	Frontal_mid_L to cerebellum_6_R	3.59
Precuneus_L to posterior insula_L	4.22	Frontal_mid_L to cerebellum_6_L2	3.42
dACC to anterior insula_L	4.19	Anterior insula_L to anterior insula_R	3.41
Cingulum_mid_R to posterior insula_L	4.15	Thalamus_L to thalamus_R	3.36
dACC to anterior insula_R	4.09	Frontal_mid_L2 to cerebellum_6_R	3.31
Cerebellum_6_L to cerebellum_6_R	4.07	Frontal_mid_L2 to cerebellum_6_L2	3.30
Anterior insula_L to supramarginal_L	4.06	Frontal_mid_R to cerebellum_6_L	3.29
Anterior insula_L to posterior insula_L	4.06	Supramarginal_L to cerebellum_6_R	3.28
Anterior insula_L to supramarginal_R	4.00	Supramarginal_R to posterior insula_L	3.26
Cingulum_mid_R to posterior insula_R	3.93	dACC to posterior insula_L	3.26
Precuneus_L to cerebellum_6_L2	3.72	Frontal_mid_R to posterior insula_L	3.23
Anterior insula_L to cingullum_mid_R	3.69	Frontal_mid_L to anterior insula_L	3.21
Cingulum_mid_R to cerebellum_6_L2	3.69	Frontal_mid_R to anterior insula_R	3.16

### Classification

**FIGURE 3:**
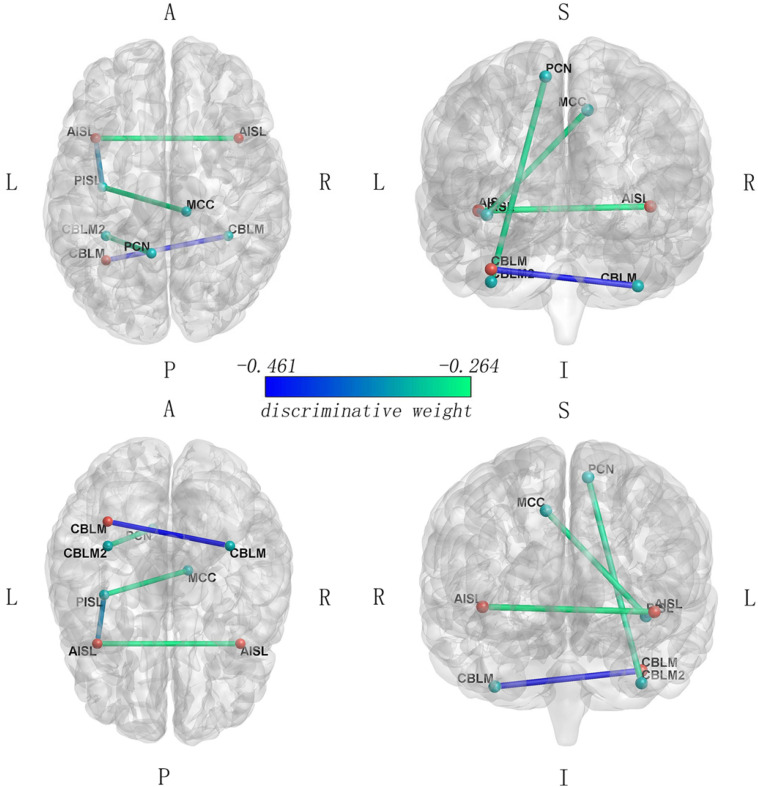
Functional connectivity with high discriminative contribution in the LSVM classifier (FC that carried greater weight with the absolute value of weight > mean + SD). The blue colors correspond to higher absolute weights in the discrimination between two groups. Red nodes, the anterior SN regions; green nodes, the posterior SN regions.

The LSVM classifier showed significant accuracy (72.09%, *p* < 0.001), sensitivity (81.40%, *p* < 0.001), and specificity (62.79%, *p* < 0.001) as well as positive (68.63%, *p* < 0.001) and negative (77.14%, *p* < 0.001) predictive values. The AUC for this analysis was 0.7820. The feature vectors, which may show high discriminative contribution in the classifier with discriminative weights values (an absolute value of >mean + 1SD, mean = 0.156, SD = 0.106), included the FC between bilateral CBLM-VI, between the left AISL and the left PISL, between the left CBLM-VI and the left PCN, between the left PISL and the right MCC, and between the bilateral AISL. Those were shown in Figure 3 and Table 3.

**TABLE 3:** The discriminative weight of feature vectors.

SN functional connectivity	MNI coordinates (x,y,z)	Discriminative weight
Cerebellum_6_L to cerebellum_6_R	(−34, −56, −32) to (36, −42, −40)	−0.461
Anterior insula_L to posterior insula_L	(−40, 14, −4) to (−36, −14, −6)	−0.357
Cerebellum_6_L2 to precuneus_L	(−34, −42, −38) to (−8, −52, 60)	−0.286
Posterior insula_L to mingulum_mid_R	(−36, −14, −6) to (12, −28, 44)	−0.281
Anterior insula_R to anterior insula_L	(42, 14, −2) to (−40, 14, −4)	−0.264
Cerebelum_6_L2 to frontal_mid_R	(−34, −42, −38) to (28, 46, 26)	−0.233
Posterior insula_L to precuneus_L	(−36, −14, −6) to (−8, −52, 60)	−0.226
Anterior insula_R to dACC	(42, 14, −2) to (0, 16, 46)	−0.216
Anterior insula_L to dACC	(−40, 14, −4) to (0, 16, 46)	−0.212
Anterior insula_R to frontal_mid_R	(42, 14, −2) to (28, 46, 26)	+ 0.178
Anterior insula_R to supramarginal_R	(42, 14, −2) to (62, −32, 42)	−0.168
Cerebelum_6_L2 to frontal_mid_L	(−34, −42, −38) to (−32, 46, 22)	−0.157
Posterior insula_R to cingulum_mid_R	(40, −6, −8) to (12, −28, 44)	−0.142
Anterior insula_L to supramarginal_R	(−40, 14, −4) to (62, −32, 42)	−0.138
Cingulum_mid_R to anterior insula_L	(12, −28, 44) to (−40, 14, −4)	−0.133
Cerebelum_6_L2 to frontal_mid_L2	(−34, −42, −38) to (−40, 36, 32)	−0.114
Posterior insula_L to dACC	(−36, −14, −6) to (0, 16, 46)	+ 0.104
Anterior insula_L to supramarginal_L	(−40, 14, −4) to (−58, −38, 36)	−0.095
Thalamus_R to thalamus_L	(12, −14, 10) to (−12, −22, 6)	+ 0.092
Cerebelum_6_L2 to cingulum_mid_R	(−34, −42, −38) to (12, −28, 44)	−0.087
Cerebelum_6_R to frontal_mid_L	(36, −42, −40) to (−32, 46, 22)	+ 0.085
Anterior insula_L to frontal_mid_L	(−40, 14, −4) to (−32, 46, 22)	+ 0.070
Cerebelum_6_R to frontal_mid_L2	(36, −42, −40) to (−40, 36, 32)	−0.067
Anterior insula_R to supramarginal_L	(42, 14, −2) to (−58, −38, 36)	+ 0.066
Posterior insula_L to frontal_mid_R	(−36, −14, −6) to (28, 46, 26)	+ 0.059
Cerebelum_6_R to supramarginal_L	(36, −42, −40) to (−58, −38, 36)	+ 0.029
Posterior insula_L to supramarginal_R	(−36, −14, −6) to (62, −32, 42)	+ 0.028
Cerebelum_6_L to frontal_mid_R	(−34, −56, −32) to (28, 46, 26)	−0.022

### Association Analysis

**FIGURE 4:**
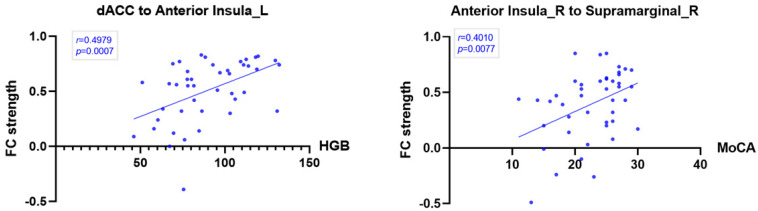
Correlation between significant SN intrinsic connectivity and hemoglobin and MoCA in ESRD patients (*p* < 0.01). The FC between the dACC and the left anterior insula was significantly positively correlated with hemoglobin (HGB). The FC between the right supramarginal gyrus and the right anterior insula was significantly positively correlated with MoCA.

The Pearson’s correlations revealed that FC between the dACC and the left AISL was significantly positively correlated with hemoglobin (*r* = 0.4979, *p* < 0.001). We also found that the FC between the right SMG and the right AISL was significantly positively correlated with MoCA (*r* = 0.4010, *p* = 0.008) by Pearson’s correlation analyses (Figure 4).

### Mediation Analysis

**FIGURE 5:**
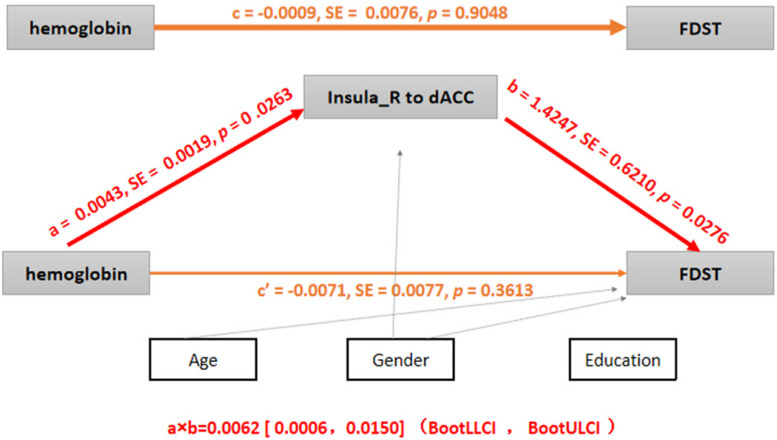
Mediation analysis (*p* < 0.05 or 95% confidence intervals did not contain zero). *c*, the total effect of hemoglobin on FDST; a, the effect of hemoglobin on FC between the right insula and dACC; *b*, the effect of FC (the right insula to the dACC) on FDST after controlling hemoglobin; *c’*, the direct effect of hemoglobin on FDST after controlling FC between the right insula and the dACC; and *a* × *b*, the indirect effect of the hemoglobin on FDST by testing whether the relationship was significantly reduced after controlling for FC between the right insula and the dACC, so *c* = *c’* + *a* × *b*. Age, sex, and education were also included as covariates, SE: standard errors, BootLLCI and BootULCI means the maximum and minimum of 95% confidence intervals using the bootstrap method.

The indirect effects existed for the FC between the dACC and the right AISL in the association between hemoglobin and FDST scores (*a* × *b* = 0.0062; 95% CI: 0.0006–0.015). But the total (*c* = −0.0009, *p* = 0.9048) and direct effects (*c’* = −0.0071, *p* = 0.3613) of hemoglobin on FDST were non-significant, suggesting that suppressing effects existed and the relationship between hemoglobin and FDST scores became significant when the FC between the dACC and the right AISL were included (MacKinnon et al., 2000). So the weaker FC between the dACC and the right AISL mediated the effect of the lower level of hemoglobin on working memory and attention deficits (Figure 5).

## Discussion

This study exclusively investigated alterations, classification ability, and mediation role of SN connectivity in ESRD patients. Our results showed abnormal and discriminative SN connectivity in patients with ESRD based on the LSVM classifier. Behaviorally, the ESRD patients exhibited both significantly global cognitive decline, mainly in working memory, attention, and processing speed, and depressive scores. And a positive correlation was observed between MoCA and FC between the right AISL and the right SMG. FC between the dACC and the left AISL was positively correlated with hemoglobin level. And the level of hemoglobin significantly affected cognitive function through an indirect path via FC between the dACC and the right AISL. Altogether, these findings suggest that SN connectivity may play a critical role in mediating the effects of anemia on cognitive impairment in ESRD.

One focus of this study was the SN due to its role on the brain end of the renal-brain axis. Our current findings were generally consistent with earlier reports, for example, recent studies have reported gray matter loss (Zhang et al., 2013), disrupted white matter microstructure (Mu et al., 2018a), elevated cerebral blood flow (CBF) (Jiang et al., 2016), and dysconnectivity (Mu et al., 2018b) in the SN in patients. The SN is engaged in identifying and responding to salient stimuli, as well as recruiting relevant large-scale functional networks for advanced cognition (Chand and Dhamala, 2016). Microscopically, most regions of the SN (i.e., the dACC and anterior insula) contain the *von Economo* neurons, which mainly appear in hominids and other advanced primates and have been suggested as a marker of successful evolution in social cognition (Allman et al., 2011). Much neuroimaging work suggests that the SN may act as a gating regulator from sensory and cognitive processing to perceptual decisions, and coordinate behavioral outputs by affecting other large-scale brain networks including the DMN and CEN (Uddin, 2015; Chand and Dhamala, 2016). A failure of regulation of activity in the DMN and CEN caused by damage to SN connectivity leads to a reduction in cognitive control and memory in aging and Alzheimer’s disease (Uddin, 2015; La Corte et al., 2016). Due to its central role in information communicating and large-scale brain network manipulating, the SN may be susceptible to homeostatic changes in the brain, thus it is the other end on the kidney-brain axis and is a marker of cognitive dysfunction.

We identified that, in ESRD patients, there were significantly reduced FC pairs between the dACC and left AISL, right AISL and right SMG, left PISL and right MCC and left PCN, and left CBLM-VI and right MFG, which was consistent with previous structural neuroimaging research (Zhang et al., 2013; Mu et al., 2018b) and a recent perspective review (Chen et al., 2015, 2017, 2018). In ESRD patients, the accumulation of uremic toxin and inflammation are important to accelerate neurodegeneration and vascular injury (Chiu et al., 2019). The AISL is particularly vulnerable to these external insults and is significantly correlated with cognitive impairment (Fathy et al., 2019). The dACC is involved in depression and the cognitive decline of ESRD (Chen et al., 2017). For efficient cognition, the FC between the dACC and the AISL can predict and regulate DMN activity (Bonnelle et al., 2012), and DMN activity is the most promising biomarker of cognitive decline in many ESRD studies (Ni et al., 2014; Luo et al., 2016). The SMG is connected to the AISL for the integration of cognition (Cauda et al., 2011; Yang et al., 2018). A hemodialysis ESRD patient research showed significantly decreased regional homogeneity in the right insula and the right SMG (Chen et al., 2015). The PISL is connected to the MCC and PCN for environmental monitoring, and response selection is based on self feeling and memory (Cavanna and Trimble, 2006; Cauda et al., 2011). The CBLM-VI contributes to emotional processing and limbic control of the motor system (Habas et al., 2009). The MFG is a part of dlPFC which is involved in cognitive planning, processing, and working memory (Fried et al., 2014). A research about hemodialysis ESRD patients reported significantly decreased FC between the right dlPFC and bilateral posterior cerebellar lobes (within fronto-cerebellar circuits) (Qiu et al., 2014).

It was interesting to find that the dACC to the left AISL connectivity was significantly positively correlated with the level of hemoglobin and that the FC between the dACC and the right AISL mediated the indirect effect of hemoglobin on FDST scores, which may confirm the relationship between anemia and cognitive decline in ESRD patients and the key role of the decreased FC in anemia-related working memory and attention deficits in ESRD. Anemia is associated with the increased risk of cognitive decline and dementia in the general population (Shah et al., 2011) and ESRD patients (Kurella Tamura et al., 2011), which can be improved after anemia treatment with erythropoietin (Hung et al., 2019). Recent ESRD patient research found weaken FC were significantly correlated with anemia and poor neuropsychological test performance (Zheng et al., 2014; Luo et al., 2016; Ma et al., 2016; Zhang et al., 2016). It may be caused by long-term hemodialysis and reduced hemoglobin could lead to cerebral dysfunction in oxygenation and regulation in CBF (Jiang et al., 2016; Zheng et al., 2016). Moreover, hemoglobin concentration may affect blood oxygen level-dependent (BOLD) signals, coupling with alterations of brain activity. Some research found the interaction between the elevated CBF and disrupted microstructure involving the ACC and insula, cognitive dysfunction, and low level of hemoglobin (Jiang et al., 2016; Mu et al., 2018a). So our findings may further deepen the understanding of the relationship between anemia and cognitive impairment from functional dysconnectivity within the SN.

The LSVM classifier had an excellent classification power with acceptable accuracy and high sensitivity and AUC, which may further prove that the SN may play a critical role in the brain function deficits of ESRD patients. A rs-fMRI research about ESRD also reported that an abnormal connectivity mode such as the left PCN, which showed acceptable accuracy and high specificity in distinguishing between ESRD patients and the HC group by receptive operation characteristic analyses (Li et al., 2016). Though there has been few studies about classifiers in ESRD patients, LSVM classifiers could predict cognitive impairment in ESRD patients referring to baseline neuroimaging data in the future.

In our study, the FC with high discriminative contribution mainly included bilateral CBLM-VI, the left AISL to the left PISL, the left CBLM-VI to the left PCN, the left PISL to the right MCC, and the bilateral AISL. The PISL is functionally connected to the MCC and sensory areas and can convey visceral sensation, whereas the AISL is mostly connected to the limbic system and can bring emotional aspect information. The interaction of the AISL and PISL is involved in modulating physiological reactivity to salient stimuli and integrating interoception and exteroception with emotion and memory and giving the feelings of self (Cauda et al., 2011; Uddin, 2015). Moreover, the insula activity can be applied to classification in autism (Uddin et al., 2013) and the prediction of disease progression in frontotemporal dementia (Day et al., 2013). The FC between the bilateral CBLM-VI contributes in emotional processing, and the FC between the left CBLM-VI and the left PCN contributes in spatial processing, working memory tasks, and executive function (Stoodley and Schmahmann, 2009). Both of them also showed high discriminative weights in the classifier for chemotherapy-treated breast cancer survivors with cognitive decline (Kesler et al., 2013).

In our study, ESRD patients reported cognitive decline, mainly in working memory, attention, processing speed, accompanied by depression, which was consistent with previous research (Dong et al., 2016). Recent rs-fMRI research also found that abnormal interaction between the SN and affective network, which laid the neural foundation of the abnormal interaction between depressive mood and cognitive control deficits in ESRD patients (Li et al., 2018; Mu et al., 2018b). And the FC between the right anterior insula and the right supramarginal gyrus significantly positively correlated with MoCA, which meant the decreased FC may predict the cognitive impairment in ESRD patients.

There are some limitations in our study. First, ESRD patients with different etiologies, and more broadly different types of diseases may be led to cognitive decline differently. Second, though we found the effects of anemia (inclined to vascular risk factor caused by kidney dysfunction) on SN connectivity and cognition in ESRD patients, we remained unsure whether the results were mainly driven by the vascular injury or neurodegenerative alterations or both of them. Third, we did not remove global signals and some specific region correlations may be covered by the effect of the low level of hemoglobin. Fourth, large samples and longitudinal data are needed to further test reproducibility of our results and determine quantitative criteria and find diagnostic and prognostic biomarkers for the treatment of cognitive dysfunction in ESRD patients.

## Conclusion

End-stage renal disease patients demonstrate dysconnectivity within the SN, which may help explain cognitive declines. The SN may serve as a potentially early biomarker in ESRD. Anemia may be an important risk factor for cognitive impairment in ESRD patients, which was mediated by decreased connectivity between the dACC and the right AISL.

## Data Availability Statement

All datasets generated for this study are included in the article/Supplementary Material.

## Ethics Statement

The studies involving human participants were reviewed and approved by the Ethics Committee of the Zhongnan Hospital of Wuhan University. The patients/participants provided their written informed consent to participate in this study.

## Author Contributions

HX, XW, RH, and LG performed the design of the investigation. RH, PC, and BW collected the subjects and applied for the ethics. RH and LG contributed to the analysis of the resting-state fMRI data and wrote the manuscript. All authors contributed to the article and approved the submitted version.

## Conflict of Interest

The authors declare that the research was conducted in the absence of any commercial or financial relationships that could be construed as a potential conflict of interest.

## Footnotes

1 http://www.rfmri.org/dpabi

2 www.fil.ion.ucl.ac.uk/spm

3 www.mathworks.com

4 http://findlab.stanford.edu/research.html

5 http://www.nitrc.org/projects/nbs/

6 http://www.csie.ntu.edu.tw/~cjlin/libsvm/
