# Supplementary material for: How Do You Feel Now? The Salience Network Functional Connectivity in End-Stage Renal Disease
Source: Front Neurosci. 2020 Nov 11;14:533910. doi: 10.3389/fnins.2020.533910 (PMC7693456; doi:10.3389/fnins.2020.533910)
Supplement: Supplementary file 1 [file Table_1.DOCX]

**Supplimentary materials**

**Table S1 Seed regions of the SN.**

|  | **cluster** | **voxels** | **Center coordinates** | | |
| --- | --- | --- | --- | --- | --- |
|  |  |  | **x** | **y** | **z** |
| **Anterior SN** | Frontal_Mid_L | 651 | -32 | 46 | 22 |
|  | Anterior Insula_L | 305 | -40 | 14 | -4 |
|  | dACC | 2887 | 0 | 16 | 46 |
|  | Frontal_Mid_R | 470 | 28 | 46 | 26 |
|  | Anterior Insula_R | 319 | 42 | 14 | -2 |
|  | Cerebelum_6_L | 95 | -34 | -56 | -32 |
|  | Cerebelum_Crus1_R | 139 | 36 | -58 | -32 |
| **Posterior SN** | Frontal_Mid_L2 | 93 | -40 | 36 | 32 |
|  | SupraMarginal_L | 1205 | -58 | -38 | 36 |
|  | Precuneus_L | 98 | -8 | -52 | 60 |
|  | Cingulum_Mid_R | 56 | 12 | -28 | 44 |
|  | Postcentral_R | 133 | 20 | -50 | 68 |
|  | SupraMarginal_R | 1002 | 62 | -32 | 42 |
|  | Thalamus_L | 142 | -12 | -22 | 6 |
|  | Cerebelum_6_L2 | 102 | -34 | -42 | -38 |
|  | Posterior Insula_L | 114 | -36 | -14 | -6 |
|  | Thalamus_R | 63 | 12 | -14 | 10 |
|  | Cerebelum_6_R | 13 | 36 | -42 | -40 |
|  | Posterior Insula_R | 134 | 40 | -6 | -8 |
